# 3D‐Printed Soft Lithography for Complex Compartmentalized Microfluidic Neural Devices

**DOI:** 10.1002/advs.202001150

**Published:** 2020-06-15

**Authors:** Janko Kajtez, Sebastian Buchmann, Shashank Vasudevan, Marcella Birtele, Stefano Rocchetti, Christian Jonathan Pless, Arto Heiskanen, Roger A. Barker, Alberto Martínez‐Serrano, Malin Parmar, Johan Ulrik Lind, Jenny Emnéus

**Affiliations:** ^1^ Department of Experimental Medical Sciences Wallenberg Neuroscience Center Division of Neurobiology and Lund Stem Cell Center BMC A11 Lund University Lund S‐22184 Sweden; ^2^ Department of Biotechnology and Biomedicine (DTU Bioengineering) Technical University of Denmark Produktionstorvet, Building 423 Lyngby 2800 Kgs. Denmark; ^3^ Department of Healthcare Technology (DTU Health Tech) Technical University of Denmark Produktionstorvet, Building 423 Lyngby 2800 Kgs. Denmark; ^4^ John van Geest Centre for Brain Repair & Department of Neurology Department of Clinical Neurosciences and WT‐MRC Cambridge Stem Cell Institute University of Cambridge Cambridge CB2 1TN UK; ^5^ Department of Molecular Biology Universidad Autónoma de Madrid and Department of Molecular Neuropathology Center of Molecular Biology Severo Ochoa (UAM‐CSIC) Nicolás Cabrera 1 Madrid 28049 Spain

**Keywords:** 3D printing, compartmentalized devices, fast prototyping, human neural stem cells, neurite guidance, nigrostriatal pathway, soft lithography

## Abstract

Compartmentalized microfluidic platforms are an invaluable tool in neuroscience research. However, harnessing the full potential of this technology remains hindered by the lack of a simple fabrication approach for the creation of intricate device architectures with high‐aspect ratio features. Here, a hybrid additive manufacturing approach is presented for the fabrication of open‐well compartmentalized neural devices that provides larger freedom of device design, removes the need for manual postprocessing, and allows an increase in the biocompatibility of the system. Suitability of the method for multimaterial integration allows to tailor the device architecture for the long‐term maintenance of healthy human stem‐cell derived neurons and astrocytes, spanning at least 40 days. Leveraging fast‐prototyping capabilities at both micro and macroscale, a proof‐of‐principle human in vitro model of the nigrostriatal pathway is created. By presenting a route for novel materials and unique architectures in microfluidic systems, the method provides new possibilities in biological research beyond neuroscience applications.

## Graphical abstract

In this study, a hybrid additive manufacturing approach to soft lithography is developed for the fabrication of open‐well compartmentalized microfluidic devices used to engineer human stem‐cell derived neural networks in vitro. The approach provides larger freedom of design, removes the need for manual postprocessing, increases the biocompatibility of the system, and enables fast prototyping at the micro and macroscale.

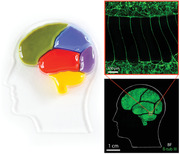

## 1 Introduction

The complexity of the human brain renders it challenging to model the number of simultaneous processes occurring in vivo at any one time. However, culturing neuronal populations in compartmentalized microfluidic devices for neurite guidance provides better means by which to deconstruct the spatiotemporal complexity of normal development and establishment of brain circuits in vitro.^[^ ^1^, ^2^ ^]^ Such compartmentalized devices have become a widely applied tool for the studying of neuronal microenvironments.^[^ ^3^ ^]^ They typically consist of two main components: microfluidic compartments for the culture of physically separated neuronal populations and microchannel array connecting the compartments that allows neurite outgrowth while preventing cell migration. Since the first experiments with the Campenot chamber were performed more than four decades ago,^[^ ^4^ ^]^ compartmentalized devices have been extensively used to study synaptic plasticity,^[^ ^5^ ^]^ axonal injury and transport,^[^ ^6^ ^]^ viral infection,^[^ ^7^ ^]^ neural circuit formation,^[^ ^8^, ^9^ ^]^ as well as disease processes.^[^ ^10^ ^]^

Microfluidic devices are most commonly fabricated using soft lithography, a prototyping technique where a soft elastomer poly(dimethylsiloxane) (PDMS) is replica‐molded from a photolithographically patterned master mold. Gas permeability, optical transparency, low cost, ease of handling, and biocompatibility are some of the advantages that have made PDMS the most frequently used material in microfluidic cell biology research.^[^ ^11^ ^]^ However, even after decades of advancements in soft lithography, PDMS microdevices and parts (e.g., for microcontact printing), are still made by firstly pouring liquid elastomer over a carefully micro or nanostructured mold and then, paradoxically, manually cutting out individual pieces by hand using a sharp tool. This step limits overall reproducibility, can damage microstructures, and cause misalignment issues. Furthermore, it limits design options. For instance, conventional soft lithography is inadequate for the realization of open compartment designs with high aspect‐ratio walls and well‐defined microstructures. Indeed, such designs have previously been sought, e.g., using hybrid mold fabrication schemes and specialized equipment, with varying degrees of success.^[^ ^12^, ^13^ ^]^

**Figure 1:**
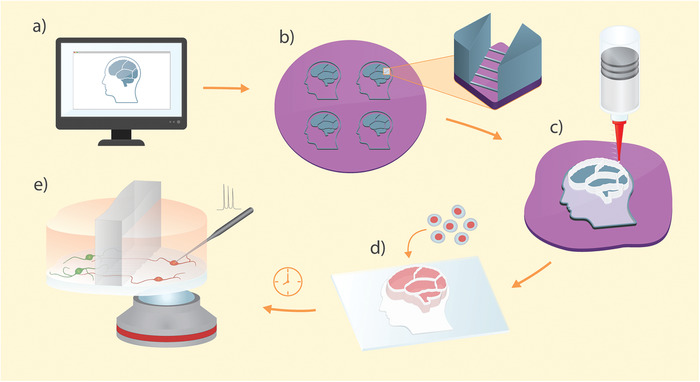
Graphical representation of the 3D‐printed soft lithography process: a) Design of the compartmentalized microfluidic device is created in a graphic editing software. b) Standard photolithography is used to create a master mold with features for the replication of both microchannels and open compartments. c) The device is made by direct 3D printing of PDMS on the master mold. d) Individual devices are peeled off the master mold, bound to a cover glass, and cells are seeded and differentiated in the compartments. e) Healthy neurons are generated on the device and allowed to interconnect through the microchannel arrays. The device design permits direct optical and physical access to the cells of interest.

In this paper we developed a novel additive manufacturing technique for the fabrication of microfluidic devices that provides a simple, yet versatile, methodology which we term 3D‐printed soft lithography (**Figure** **1**). The method retains the advantages of the conventional soft lithography while it minimizes manual postprocessing of the device, expands design possibilities, simplifies the fabrication of high aspect ratio features, and allows reduction of the PDMS content through controlled deposition of the elastomer. Furthermore, 3D‐printed soft lithography allows fast prototyping with precisely defined features across scales and opens up possibilities for the introduction of new materials to the soft lithography toolbox. The 3D‐printed devices provide a platform for long‐term differentiation and maintenance of human stem‐cell derived neurons and astrocytes as well as neurons directly reprogrammed from adult human fibroblasts (or for that matter, any cell cultures). To show the potential of the novel fabrication approach, we undertook a proof‐of‐principle experiment in which we modeled the nigrostriatal pathway using human neural stem cells. The degeneration of dopaminergic neurons in this pathway is the hallmark of Parkinson's disease (PD) and causes the characteristic motor symptoms associated with it. An important feature of the nigrostriatal pathway is the directionality of dopaminergic projections from ventral midbrain to dorsal striatum in the forebrain. By utilizing fast prototyping approach for microstructure design, we engineered microchannels to maximize the unidirectional growth of dopaminergic projections between two compartments. The developed device does not only provide a platform to study PD in vitro but also exhibits the versatility of 3D‐printed soft lithography in neuroscience.

## 2 Results

### 2.1 3D‐Printed Soft Lithography

**Figure 2:**
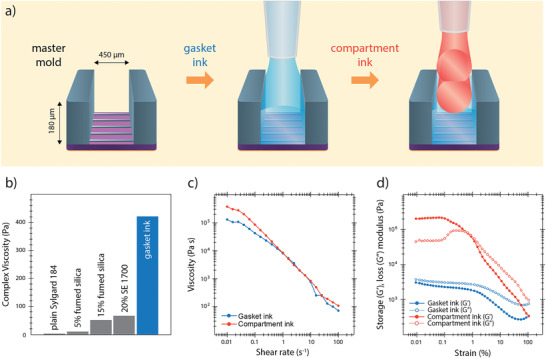
Rheological properties of the inks: a) Illustration showing the deposition of the two inks on the master mold (not to scale). A flowing gasket ink is printed directly on top of the master mold. The ink covers the small features for the molding of the microchannels but does not flow over the tall features that mark the compartment borders creating defined vertical edges. Multiple layers of compartment ink are deposited on top of the gasket to fabricate compartment walls. b) The complex viscosity at 0.01% strain increases as additives are mixed with the Sylgard 184 base. The finally used gasket ink includes 15% fumed silica and 20% SE 1700. c) Decrease in viscosity with higher shear rates under steady‐state conditions indicates shear thinning behavior for both inks. d) Storage modulus (*G*′) and loss modulus (*G*′′) as a function of applied strain amplitude in an oscillatory sweep mode. The compartment ink undergoes solid‐to‐liquid transition illustrated by the crossover around 1% strain. On the other hand, gasket ink is dominated by liquid properties across the whole range of strain amplitudes.

PDMS is 3D‐printed in two steps directly onto the silicon wafer containing the master mold pattern by pressure driven extrusion through a syringe nozzle (**Figure** **2**a). Two inks were formulated for this purpose: a flowing “gasket ink” for high fidelity molding without entrapment of air bubbles and a “compartment ink” for the fabrication of vertical structures. Inks were created by blending two types of commercially available elastomers with very different rheological properties (Sylgard 184 and Dowsil SE 1700). Sylgard 184 is a liquid elastomer widely used in conventional soft lithography, whereas SE 1700 is a shear thinning PDMS elastomer with well suited material properties for 3D printing applications, including open well structures.^[^ ^14^ ^]^

The gasket ink was created by modulating the rheology of Sylgard 184 as the base component. In plain form, this elastomer behaves as a Newtonian fluid without sufficiently high viscosity to be controllably extruded through a nozzle and to support the weight of layers printed on top of it. The desired increase in viscosity was achieved by the addition of fumed silica nanoparticles and SE 1700 (Figure 2b). The nanoparticles, made of amorphous silicon dioxide, interact with each other through hydrogen bonds and form a loose network that modifies the rheology of the elastomer.^[^ ^15^ ^]^ At the optimal formulation, the complex viscosity of the ink at low strains is increased 100‐fold (from 4 to 420 Pa) compared to the pure Sylgard 184 elastomer. Unlike pure Sylgard 184, the gasket ink shows shear thinning behavior (Figure 2c), a necessary feature for the controllable and consistent flow during printing. Most importantly, the loss modulus of the gasket ink at low strain rates is higher than the storage modulus indicating that it has predominantly liquid‐like properties at rest (Figure 2d). This characteristic of the gasket ink allows it to flow after it is deposited and uniformly cover the features on the master mold (Figure 2a middle).

The compartment ink was formulated for the fabrication of structurally stable high‐aspect ratio vertical features. The base of the compartment ink is SE 1700 elastomer which by itself is a suitable material for 3D printing. Addition of Sylgard 184 to the final formulation enhances the printing properties of the ink.^[^ ^16^ ^]^ The ink exhibits shear thinning behavior indicating its suitability for extrusion‐based 3D printing with high accuracy (Figure 2c). Unlike the gasket ink, the compartment ink is dominated by solid‐like properties at rest with the elastic modulus being higher than the loss modulus at low strains (Figure 2d). As a result, the ink retains its shape once it is deposited maintaining the structural integrity of the compartment walls.

**Figure 3:**
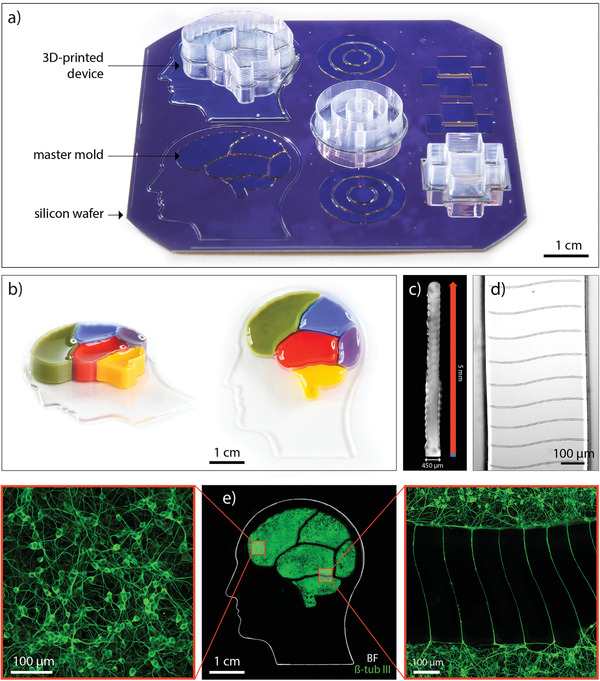
Fabricating devices with complex designs: a) Image shows the silicon wafer containing the master mold for three device designs: “brain” device, concentric circles device, and axotomy device. One device for each design is fully 3D‐printed while the other molds are left empty in order to visualize the starting point compared to the final product. b) Photographs of the fabricated device. Colored inks were poured into each compartment to demonstrate physical separation between individual compartments. c) Inverted brightfield image of a compartment wall cross‐section. The blue part of the dimension line indicates the part of the wall printed with the gasket ink. The red part indicates the ribbed part of the wall printed with the compartment ink. d) Brightfield image of the patterned wavy microchannels on the bottom face of the compartment wall. e) Middle: image composed of an inverted brightfield image illustrating the edges of the gasket resembling a human head and a fluorescent image indicating the extent of the neuronal network in each compartment marked by *β*‐tubulin III, a microtubule element found almost exclusively in neurons; left: higher magnification fluorescent image of the neurons in the middle of a compartment; right: higher magnification fluorescent image of the neurons extending projections through microchannels.

To explore the fabrication capabilities of 3D‐printed soft lithography, we next generated several compartmentalized microfluidic devices with arbitrary complex designs: a “brain” device, a concentric circle device, and an axotomy device (**Figure** **3**a). The fabrication of these devices is not possible with conventional soft lithography and highlights the potential of 3D‐printed soft lithography for the manufacturing of multiscale features with high‐aspect ratio vertical compartment walls.

As a first example, we made a 4 × 4 cm compartmentalized “brain” device with defined structural elements both at micro and macroscale (printing of the device is shown in Video S1, Supporting Information). The gasket of the device resembles the outline of the human head while the five compartments for neuronal cultures resemble five regions of the human brain. Compartments were loaded with inks of different colors in order to demonstrate physical separation between them (Figure 3b). Each compartment is enclosed within 5 mm tall walls allowing spatial separation of cell bodies. The walls are vertical with width of 450 µm and height of 5 mm (Figure 3c). Such high aspect ratio (11:1) was achieved by printing the compartment ink through a 200 µm nozzle with a single layer width. Individual layers can be identified in the ribbed texture of the walls. As can be seen in Figure 3d, microchannels are faithfully replicated from the mold on the bottom face of the compartment walls. 4.7 µm high and 8 µm wide, the microchannels span the whole width of the wall connecting the two adjacent compartments.

To indicate the biological suitability of the fabricated device (Figure 3e middle), human neural stem cells (hNSCs) were differentiated in the compartments generating a dense neuronal network that covers the bottom surface of each compartment (Figure 3e left). Microchannels then allow for neurite outgrowth connecting different regions of the “brain” device (Figure 3e right).

The second design that exemplifies the ability of the developed method, the concentric circle device, is made to maximize the microchannel interface between the compartments and incorporates a “return‐to‐sender” microchannel design that has been shown to induce directionality in neurite outgrowth (Figure S1a–c, Supporting Information).^[^ ^17^ ^]^

The third design, the axotomy device, exemplifies the suitability of 3D‐printed soft lithography for the fabrication of closed microfluidic channels. Besides the two open compartments, this device contains a closed microfluidic axonal compartment which can be used for the severing of neural projections for the study of axonal injury and regeneration (Figure S1d–f, Supporting Information).

Due to the controlled deposition of PDMS, there is no need for manual postprocessing (e.g., cutting with a scalpel or a biopsy punch) of devices that can cause damage to the microstructures or create unwanted overhangs (Figure S2, Supporting Information). After the printing is done, fabricated devices are simply cured in the oven, peeled off the master mold, and bound to a cover glass. 3D‐printed soft lithography could also be used for more accurate and reproducible fabrication of stamps for microcontact printing where the positioning of the micropatterns with respect to the outer boundaries of the stamp is a known issue (Figure S3, Supporting Information).

### 2.2 Biocompatible Compartmentalized Devices for hNSC Differentiation

**Figure 4:**
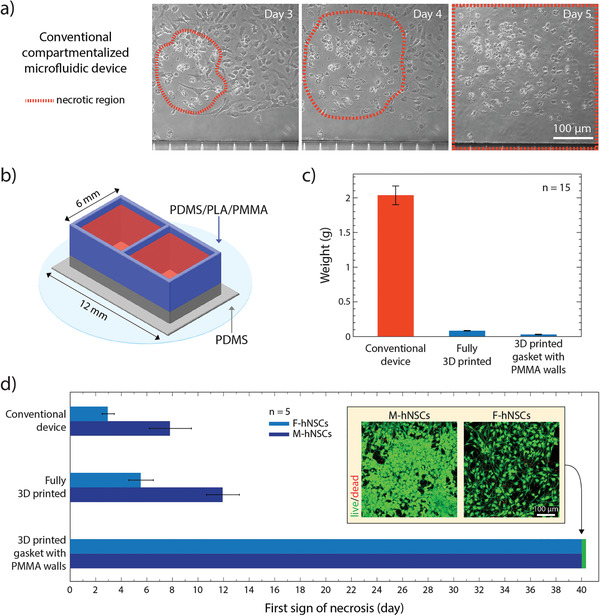
Long‐term culture of hNSCs in 3D‐printed devices: a) Brightfield image of the necrotic core identified in the F‐hNSC population 3 days after the differentiation was initiated in a conventional compartmentalized PDMS device. On day 5 of differentiation, the necrotic region expands throughout the whole population. b) Graphical illustration of a two‐compartment device used in the cell viability study. c) Comparison of PDMS content between the conventional device and the devices made using 3D printed soft lithography. d) A bar chart displaying the first recorded observation of the necrotic region for each hNSC line in compartmentalized devices with decreasing ratios between the cell media and the PDMS surface area exposed to the media (mean value with standard deviation). The insert shows live/dead staining of the cells after 40 days of differentiation in the device with PMMA extensions.

Two different hNSC lines were used in this study. One was made from the forebrain region of a human embryo and is hereafter referred to as F‐hNSC. The second was derived from the ventral mesencephalon in the midbrain and is referred to as M‐hNSC. When differentiated in a conventional well plate, F‐hNSCs generate a cell population with a high percentage of astrocytes while M‐hNSCs generate a high percentage of dopaminergic neurons.^[^ ^18^, ^19^ ^]^ Our initial experiments with conventional PDMS‐based microfluidic compartmentalized devices proved to have detrimental effects on both cell lines (**Figure** **4**a). At a certain point during differentiation, cell death was observed as localized necrotic kernel regions, which increased in size and eventually spread throughout the whole cell population. F‐hNSCs were particularly sensitive to this phenomenon. As can be seen in Figure 4a, this was evident as early as day 3 in vitro. Within a couple of days necrosis expanded throughout the whole cell population. As a result, it was not feasible to generate astrocytes from F‐hNSCs. We took advantage of the sensitivity of F‐hNSCs to the environmental perturbations as a robust readout to explore the biocompatibility of the developed devices.

First, in order to decouple the effects of PDMS from the influence of the microfluidic architecture, we differentiated both F‐hNSCs and M‐hNSCs in XonaChips, a commercial injection‐molded two‐compartment device made from cyclin olefin copolymer with the same design as the conventional compartmentalized PDMS device.^[^ ^20^ ^]^ In this device differentiation was successful indicating compatibility of both cell lines with the microfluidic environment (Figure S4, Supporting Information). However, when we attempted to differentiate the F‐hNSCs in a commercial polystyrene 48‐well plate using media that was preconditioned in PDMS wells, the same pattern of necrosis, as observed in a conventional PDMS device, occurred within the first week of differentiation (Figure S5, Supporting Information). These results indicate that it is the PDMS itself that affects the cells and not the microfluidic environment.

One of the causes of the toxicity could be the presence of uncured PDMS oligomers that leach out of device walls and get embedded into cell membranes.^[^ ^21^ ^]^ It has been reported that extensive curing times and solvent extraction of unpolymerized oligomers and metal catalysts used for curing the elastomer enhance biocompatibility of the material.^[^ ^22^ ^]^ In our case, solvent extraction resulted in 4.6 ± 0.1% (*n* = 6) weight reduction in the conventional PDMS devices indicating clearing of a large amount of unpolymerized oligomers. Matrix‐assisted laser desorption/ionization–time‐of‐flight (MALDI–TOF) analysis of leached PDMS content showed the presence of PDMS oligomers in the media of conventional devices when the curing time was limited to 2 h while no oligomers were detected when curing was extended to 24 h (Figure S6, Supporting Information). However, neither solvent extraction nor extensive curing resulted in a drastic improvement in F‐hNSC survival time during differentiation suggesting that the residual presence of unpolymerized elastomer could significantly affect cell viability. Furthermore, solvent extraction is a long and laborious process that increases the device fabrication time by more than a week and introduces the risk of toxic effects from the unwashed solvent molecules.

Nonspecific absorption of proteins and small molecules is another potential drawback of PDMS devices that could affect cell differentiation and survival.^[^ ^23^ ^]^ The porous nature of the elastomer network allows the absorbed molecules to diffuse into the bulk polymer.^[^ ^24^ ^]^ As a result, the composition of the cell media could be significantly altered. The absorption is time‐dependent, nonlinear, and variable between different compounds.^[^ ^25^ ^]^ Therefore, a simple change in the composition of cell culture media would not solve the problem.

For both nonspecific absorption of molecules and leaching out of uncured elastomer, the common limiting factor is the ratio between the total cell media volume to the total volume of PDMS in a device. The amount of bulk polymer in the device acts as a reservoir for the absorbed molecules and uncured oligomers and influences the final equilibrium concentration established between PDMS and cell media. The rate limiting factor that affects the time before the equilibrium is reached is the surface area of PDMS exposed to the cell media. The exposed surface area dictates the rate at which both the oligomers leach out and the molecules from the media are absorbed into the bulk of the device.

We hypothesized that a major reduction in the total amount of PDMS in a device and the PDMS surface to media volume ratio would remove the toxic effect and allow culturing and differentiation of hNSCs in the same conditions as in a commercial polystyrene tissue culture well plate. We took advantage of 3D‐printed soft lithography to make devices with these specifications. To do so, we designed a rectangular device with two open compartments and 450 µm thin walls (Figure 4b). The device was either fully printed or, to further decrease the amount of PDMS and show how the 3D‐printing approach could be combined with other materials, 0.5 mm high walls were printed and then extended with micromilled poly(methyl methacrylate) (PMMA) compartments or 3D‐printed polylactic acid (PLA) compartments. The conventional PDMS device, the fully printed PDMS device, and the devices with PMMA or PLA extension can be seen in Figure S7 in the Supporting Information. After the fabrication, the devices were weighed (Figure 4c). Fully printed device reduces the total amount of PDMS roughly 20 times compared to the conventional device while the PMMA‐extended device introduces a 60‐fold reduction in PDMS content. The ratio between the cell media volume and the PDMS surface area exposed to cells was calculated to be 0.09, 1.5, and 15 for the conventional device, fully printed device, and PMMA‐extended device, respectively. This implies that there is a 170‐fold increase in media volume per surface area unit of exposed PDMS for the PMMA‐extended device compared to the conventional device, which should greatly minimize the effects of PDMS absorption and dilute any unpolymerized oligomers to levels below the detrimental threshold. Indeed, as can be seen in Figure 4d, the decrease of the total content and exposed surface area of PDMS in the device had a remarkable effect on the hNSC survival during the differentiation process. While fully 3D‐printed devices showed an improvement over the conventional microfluidic devices (albeit not sufficient for long term cultures), when differentiated in devices with PMMA extensions, neither cell lines showed any signs of PDMS‐specific necrosis even after 40 days in culture (Figure 4d inset). At this time point, the experiments were stopped given a proof of principle of increased biocompatibility for these sensitive cell lines had been shown. In the process, we also considered fully printed alternatives based on styrene–isoprene–styrene (SIS) block copolymer inks (Figure S8, Supporting Information). However, compared to PMMA/PDMS hybrid devises, these often detached from supports, and appeared less biocompatible. Consequently, printed PDMS devices with PMMA extensions were chosen for the subsequent experiments.

### 2.3 Generation of Functional Human Neurons and Astrocytes in 3D‐Printed Devices

**Figure 5:**
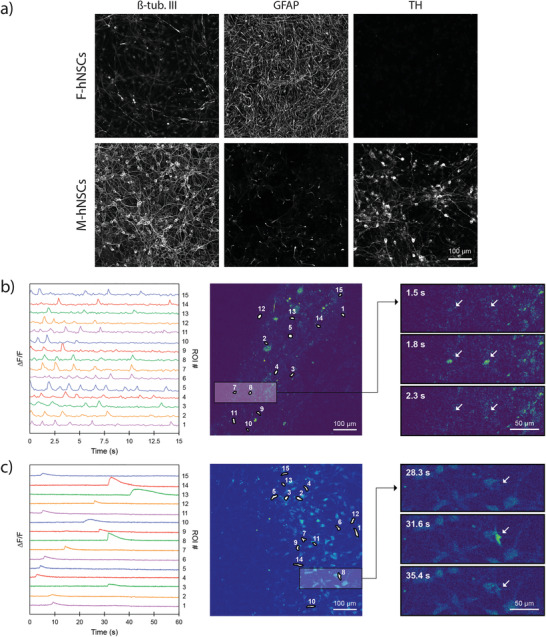
Functional neurons and astrocytes in 3D‐printed compartmentalized devices: a) Immunocytochemistry after 22 days of differentiation in a 3D‐printed compartmentalized device with a PMMA wall extension. b) Differential fluorescence intensity profile as a function of time for M‐hNSCs expressing MAP2‐GCamP3 (left); fluorescence image with segmented regions of interest corresponding to individual cells (middle); three timeframes displaying the change in intercellular fluorescence intensity for two cells indicated by arrows (right). c) Differential fluorescence intensity profile as a function of time for F‐hNSCs loaded with Fluo3AM (left); fluorescence image with segmented regions of interest corresponding to individual cells (middle); three timeframes displaying the change in intracellular fluorescence intensity for a single cell indicated by an arrow (right).

Immunocytochemistry showed that the hNSC differentiation in the PMMA‐extended devices was successful and not hindered by environmental factors. **Figure** **5**a shows high expression of neuronal marker *β*‐tubulin III in the cell population differentiated from M‐hNSCs while the same is not present in cells differentiated from F‐hNSCs. F‐hNSC derived cells are on the other hand rich in GFAP, an intermediate filament protein expressed mainly in astrocytes. Additionally, tyrosine hydroxylase (TH), a rate limiting enzyme in the synthesis of dopamine, is present in cells derived from M‐hNSCs while it is not present in those derived from F‐hNSCs. These results show the ability of our new system to allow NSCs to differentiate into astrocyte rich cell populations from F‐hNSCs and to differentiate into dopaminergic neurons from M‐hNSCs, which is well in line with the previously published results using conventional polystyrene tissue culture well plates.^[^ ^18^, ^19^ ^]^ Additionally, to show that our devices are not compatible only with human neural stem cell lines, we conducted direct conversion to generate induced neurons (iNs) from adult human patient derived fibroblasts ^[^ ^26^ ^]^ in the devices and managed to culture them for 30 days without detrimental effects (Figure S9, Supporting Information).

To demonstrate the functional activity of the generated neurons and astrocytes, we performed calcium imaging. Two approaches were followed in order to differentiate neuronal and astrocytic calcium activity. In the first approach, we expressed GCaMP3, a genetically encoded calcium marker under the MAP2 promoter, in order to visualize intracellular calcium activity specifically in neurons. As can be seen in Figure 5b, spontaneous calcium transients were recorded in the absence of any depolarizing stimuli in the neuronal population generated from M‐hNSCs. This is a sign of a healthy cell population. The transients indicate multiple spiking behavior for individual cells with spike duration of 100–300 ms and an average interval of 1.1 ± 0.4 s (Figure 5b left). Also, neuronal network generated from M‐hNSCs responds to the chemical stimulation (Video S2, Supporting Information). In contrast, there was no calcium activity observed in the astrocyte rich cell population generated from F‐hNSCs. To record activity within this population, we loaded the cells with a membrane permeable fluorescent Ca^2+^ indicator which is not cell type specific. This time, spontaneous calcium activity was recorded in the astrocyte rich population (Figure 5c left). Recorded Ca^2+^ peaked with an average delay of 0.96 ± 0.58 s following an exponential decay ranging from 5 s to more than 15 s. The dynamics of the Ca^2+^ transients in the F‐hNSC population significantly differed from the recordings made in the M‐hNSC population and resembled the astrocytic footprint of Ca^2+^ transients recorded in vivo. ^[^ ^27^, ^28^ ^]^ These results demonstrate that 3D‐printed soft lithography can be used to make microcompartment devices compatible with human stem cell differentiation into healthy functional neurons and astrocytes.

### 2.4 Reconstructing Directionality in the Nigrostriatal Pathway via Fast Prototyping of the Microscale Features

**Figure 6:**
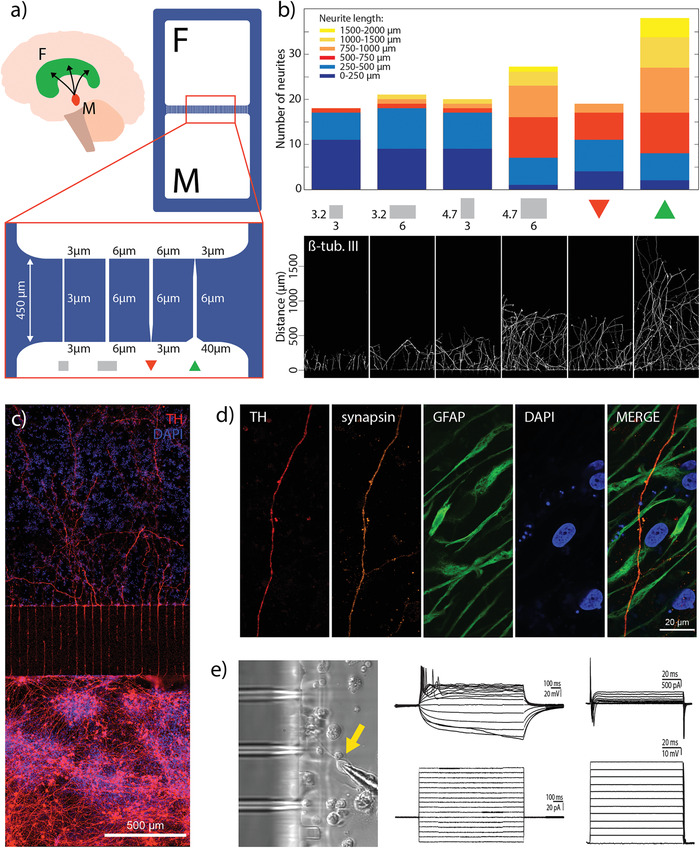
Reconstruction of the nigrostriatal pathway on‐a‐chip: a) Graphical representation of the nigrostriatal pathway that unidirectionally connects the dopaminergic neurons of the SNpc in the midbrain (M) to the dorsal striatum in the forebrain (F); graphical representation of the compartmentalized device used in this section with the illustration of different microchannel designs. b) Graph showing the number of transmitted axons and their length in the adjacent compartment for an array of 20 microchannels (*n* = 3) (top) and the corresponding fluorescent images of transmitted projections (bottom). c) Fluorescent image showing directional growth of dopaminergic axons in the adjacent compartment innervating the opposite cell population. d) Fluorescent images showing the transmitted dopaminergic axon in the opposite compartment surrounded by astrocytes. Synapsin is co‐expressed with TH on an axon transmitted in the adjacent compartment indicating the presence of synaptic vesicles. e) Brightfield image showing the micropipette tip accessing a cell in the vicinity of the microchannels (left) Whole cell patch clamp recording, showing multiple induced action potentials (upper middle) upon steps of current injection (lower middle). In Voltage clamp studies, cells displayed the presence of both Inward Sodium and Outward Potassium currents (upper right) when steps of voltages where applied (lower right).

The nigrostriatal pathway is a dopaminergic pathway that connects the *Substantia nigra pars compacta* (SNpc) in the midbrain with the dorsal striatum in the forebrain (**Figure** **6**a). This dopaminergic connection is unidirectional with the dopaminergic neurons projecting from the SNpc and delivering dopamine to the dorsal striatum. It is the loss of the dopaminergic neurons in the nigrostriatal pathway that is the hallmark of PD. Furthermore, there is mounting evidence suggesting that astrocytes could play initiating pathogenic role in PD. It has been found that PD‐related genes are expressed in astrocytes and their dysfunction could lead to neuronal toxicity.^[^ ^29^ ^]^ These findings indicate that astrocytes might be important in maintaining functional activity of dopaminergic neurons in the nigrostriatal pathway. To explore the capabilities of 3D‐printed soft lithography as a fast prototyping method by which to study neurite growth through engineered microchannels, we constructed a proof‐of principle nigrostriatal pathway on‐a‐chip. We utilized the two‐compartment device from the previous section in order to spatially separate dopaminergic neurons (representing midbrain) from astrocytes (representing forebrain) while facilitating directional outgrowth of dopaminergic projections through engineered microchannels. The architecture of the microchannels was optimized to maximize the number and length of dopaminergic axons protruding to the adjacent compartment while preventing projections in the opposite direction, mimicking the in vivo situation. Firstly, two lengths of microchannels were tested, 450 and 900 µm. As neural projections did not successfully transverse the full length of the longer microchannels (Figure S10, Supporting Information), we kept microchannel length at 450 µm in subsequent experiments. Next, we explored how the design of the microchannels affects the axonal outgrowth generated from M‐hNSCs. Although work in this field has been reported in the literature, most of it was performed with primary murine neurons.^[^ ^8^, ^17^ ^]^ Importantly, the growth dynamics and developmental pathways underlying this process are different for human dopaminergic neurons investigated here. Therefore, in our study, we could not directly apply the designs reported for murine cells. Instead, we screened 24 different microchannel designs by varying the following parameters: microchannel height, shape of the entrance into the microchannel, width of the middle section of the microchannel, and the shape of the exit from the microchannel (Figure 6a). We differentiated cells in one compartment and observed growth of neural projections in the opposite empty compartment. We identified six designs that showed the effects of the microchannel dimensions on the outgrowth of M‐hNSC‐derived neural projections (Figure 6b). 3.2 µm height hindered the outgrowth regardless of the width of the channel with only 12% of axons extending more than 500 µm into the adjacent compartment. Increasing the microchannel height to 4.7 µm but keeping the width at 3 µm did not improve neurite outgrowth. However, when the microchannel dimensions were raised to a width of 6 µm and a height of 4.7 µm, we observed a 150% increase in the number of transmitted axons with 75% extending more than 500 µm into the adjacent compartment (Figure 6b). Microchannels with larger dimensions did not effectively prevent cell migration between the compartments. Finally, we explored the effect of funnel design, also known as an axonal diode, on axonal transmission.^[^ ^30^, ^31^ ^]^ 40 µm widening at the entrance to the channel increases the probability of a neurite entering the channel while a 3 µm constriction has the opposite effect (Figure 6a red and green triangle, respectively). The results indicate that both the number of the growing axons and their length is maximized when the wide opening is included in the design. The axons protrude up to 2 mm into the adjacent compartment (Figure 6b green triangle). It is important to note that, in a separate set of experiments, when the microchannel design was screened for F‐hNSCs (that do not project along the nigrostriatal pathway), no projections were seen in the adjacent compartment from the differentiated cells (Figure S11, Supporting Information).

After the microchannel design had been optimized for the generation of an oriented neuronal network, we differentiated M‐hNSCs in the bottom compartment of a device and F‐hNSCs in the top compartment. Figure 6c displays the neuronal network generated in the device after 20 days of differentiation. The bottom compartment is rich in dopaminergic neurons with projections that extend through the microchannels and innervate the adjacent cell population. Dopaminergic projections extend deep into the opposite compartment, up to 2 mm, as expected with the chosen microchannel design. Synapsin is expressed in the dopaminergic projections indicating the presence of synaptic vesicles in the adjacent compartment, a key component for functional neuronal connections between the compartments (Figure 6d). In the same figure, a network of astrocytes can be observed surrounding the transmitted dopaminergic projection.

Besides optical access for microscopy, thin vertical walls of the developed device enable direct physical access to the cells in the vicinity of the microchannels, allowing electrophysiological whole cell‐patch clamp investigation of the differentiated hNSCs in the engineered nigrostriatal pathway (Figure 6e left). With the use of microelectrodes for single‐cell electrophysiological recordings we targeted neuronal‐like cells in the cultures. By monitoring the passive membrane properties, we found that cells retained a resting membrane potential ranging from −30 to –45 mV, indicating that the cells are successfully differentiating towards the relevant neuronal phenotype. The membrane resistance, a value dependent on the density of ion channels in the membrane, was similar among all the patched cells. Also, the size of the cells, indicated by the membrane capacitance, was similar among the patched cells. In the current clamp mode, steps of currents were injected in the cells in order to detect their ability to generate action potentials (AP) (Figure 6e middle). All the recorded cells showed the presence of induced APs as reported before for these cells in commercial tissue culture dishes.^[^ ^32^ ^]^ These recordings indicate the ability of the cells to communicate and generate electrical signals resulting from membrane depolarization. In the voltage clamp mode, we checked the presence of inward sodium and outward potassium currents as a measure of the expression of voltage‐gated sodium and potassium channels (Figure 6e right). All the cells showed such currents in the membrane. This data confirms that the cells are electrophysiologically active highlighting the potential of the developed device for modeling and studying neuronal communication.

## 3 Discussion

The hybrid additive fabrication approach presented in this paper overcomes several limitations associated with the conventional soft lithography systems based on bulk PDMS casting. In particular, it removes the need for manual cutting of the compartments using, e.g., blunt needles or biopsy punches, which is laborious and may lead to damage or misalignment of microstructures. Further, our procedure allows for a much broader range of device designs, such as high‐aspect ratio open‐compartment microfluidic systems. A few recent reports have presented alternative methods aimed at overcoming the limitations associated with such conventional devices. For instance, one report combined cast PDMS with computer numerical control (CNC) milling and hot embossing to create desired features in a PMMA master mold ^[^ ^12^ ^]^ while another extended the features on the master SU‐8 mold using 3D‐printed steel inserts.^[^ ^13^ ^]^ Compared to these procedures, the 3D‐printed soft lithography route presented here introduces a simple yet versatile and reproducible process flow. It simultaneously remains fully compatible with master molds that are used in conventional soft lithography and which are fabricated with standardized cleanroom processes for SU‐8 photolithography. Additionally, 3D‐printed soft lithography provides a route for the use of other materials, not compatible with bulk casting, for molding of microfluidic device. Stereolithography, another increasingly popular fabrication approach, has been used to produce high aspect ratio microfluidic devices but its resolution is currently limited to 18 µm for microfluidic channels and as such could not be utilized for neuroscience applications to create microchannels needed for neurite guidance with design variations on the order of a single micrometer.^[^ ^33^ ^]^

Besides the expansion of design capabilities, our procedure allows many attractive material properties of PDMS to be exploited while removing possible drawbacks related to biological applications by limiting the total amount of PDMS in a functional device. Leveraging this advantage, we were able to create compartmentalized devices for long‐term culture of human stem cell‐derived neurons and astrocytes. Notably, both cellular phenotype and viability was fully sustained for at least 40 days. This demonstrates that, while PDMS in excessive amounts may cause harm to delicate cell cultures, we can mitigate these to the point of allowing long‐term culture and studies on highly sensitive hNSCs. This accomplishment is important because the long term cultures (up to at least two weeks) of human stem‐cell derived neurons have been demonstrated so far only in several reports while the large volume of work has been performed on the primary murine neurons that are more robust and require shorter culture times.^[^ ^34^, ^35^, ^36^ ^]^ Notably, our findings also indicate that elastomers such styrene–isoprene–styrene block copolymer inks, which are less used for in vitro biology, but could be considered as alternatives to PDMS in our procedure, are not unproblematic. As alternative to lithography‐based devices, commercially available injection molded devices could be an appropriate solution for sensitive neuronal cultures.^[^ ^20^ ^]^ However, such devices are costly in small quantities and do not offer design freedom and rapid prototyping capabilities that 3D‐printed soft lithography has to offer.

Finally, we demonstrated the versatility of our procedure by creating a proof‐of‐concept culture system which allowed us to reconstruct the nigrostriatal pathway in the device. This was done by screening for microchannel designs that maximized the growth of dopaminergic projections from hNSC‐derived midbrain neurons. The results indicate how important fast prototyping and design flexibility are when creating devices for explorative studies with human cells. Alterations of the microchannel shape and dimensions in the micrometer range had a significant impact on the outgrowth of the projections, indicating further the importance of preserving intact microstructures during the fabrication process. The developed system demonstrates that 3D‐printed soft lithography allows design flexibility covering the single micrometer to centimeter range allowing adaptation of the device to specific purpose and specific cell type. We demonstrated only one scenario in which the role of astrocytes in the protection of dopaminergic neurons can be studied. Possible alternatives could be replacing astrocytes with GABAergic neurons which are inhibited by the activity of dopaminergic neurons in vivo, to study signal propagation. A system could also be designed to study transmission of *α*‐synuclein aggregates, a well‐known pathology of PD. In a bigger picture, together with stem cell technology, direct reprogramming protocols, and gene editing tools, our approach opens up a host of opportunities in neuroscience to study neurological diseases, neural network formation, and test drugs in vitro.

## 4 Conclusion

In summary, we demonstrated an additive manufacturing approach to soft lithography that allows simple fabrication of the open‐well compartmentalized devices with vertical walls and control over both microscale and macroscale features. Compared to the conventional soft lithography, our approach expands the design possibilities, overcomes the issue of inaccuracy and damage caused by manual postprocessing, and allows an increase in the biocompatibility of the system. Leveraging the advantages of the developed approach, we created compartmentalized devices that support differentiation of sensitive hNSCs into healthy neurons and astrocytes and their long‐term maintenance. Furthermore, we engineered an in vitro proof‐of‐concept model of the nigrostriatal pathway. Our approach relies on affordable commercial materials, conventional photolithography protocols, and 3D printing technology that is an increasingly common part of many research institutes. Due to its simplicity, versatility, and accessibility, 3D‐printed soft lithography has a potential to become a frequently used solution for the fabrication of microfluidic devices.

## 5 Experimental Section

##### Device Design and Master Mold Fabrication

Device elements were designed in Adobe Illustrator and further processed in Tanner L‐Edit software. Silicon wafers with thermally grown silicon oxide were used as substrates for master fabrication. As a first layer, the pattern for the molding of microchannels was fabricated using SU‐8 photolithography (for 4.7 µm tall microchannels) or reactive ion etching (for 3.2 µm tall microchannels). A second 180 µm high layer for the molding of the compartments was then fabricated using SU‐8 photolithography.

Patterning 4.7 µm Tall Microchannel Mold—SU‐8 2005 was spin coated at 5000 rpm for 60 s on a RCD8 T spin coater (Süss MicroTec) to obtain a 4.7 µm thick layer. The wafers were then soft baked for 3 min at 90 °C on a hot plate. The pattern for the microchannels was exposed using a maskless aligner (MLA100, Heidelberg Instruments). Post exposure bake was then performed for 3 min at 90 °C. This was followed by development in a dedicated bath containing the developer mr‐Dev 600 for 10 min. The wafers were thoroughly rinsed using isopropanol.

Patterning 3.2 µm Tall Microchannel Mold—A 1.5 µm negative resist (nLOF 2020) was spin coated and baked at 110 °C to act as an etch mask. The microchannel pattern was exposed using a maskless aligner. The wafers were baked post exposure at 110 °C and developed. A hard bake at 110 °C followed development to allow for the evaporation of any solvent and harden the resist for dry etching. The silicon oxide layer was first completely etched in advanced oxide etcher (STS MESC Multiplex ICP) using fluorine chemistry. This was followed by 1 min silicon etch to define the depth of the microchannel pattern at 3.2 µm. The resist mask was then stripped in oxygen.

Patterning 180 µm Tall Compartment Mold—A 180 µm thick resist layer of SU‐8 2075 was spin coated followed by a soft bake at 50 °C for 6 h. The compartment pattern was then exposed using a maskless aligner. The post exposure bake was performed at 50 °C for 6 h to allow crosslinking of the exposed SU‐8 structures. The wafers were then developed as before for 20 min and thoroughly rinsed in isopropanol. Finally, a hard bake was performed at 90 °C for 15 h.

The masters were then subjected to surface modification using 1*H*,1*H*,2*H*,2*H*‐perflourodecyltrichlorosilane (FDTS) to facilitate the removal of cured PDMS from the mold. The height of the structures and their fidelity was verified using a stylus profilometer and an optical microscope. Finally, the wafers were diced for better fitting and alignment on the printing stage.

##### Ink Formulation, 3D Printing, and Device Assembly

To create the stock for the gasket ink, fumed silica nanoparticles (200–300 nm average particle size, Sigma‐Aldrich) were gradually added to the Sylgard 184 elastomer (Dow Corning) until a 15% fumed silica content was reached. ≈1 g of nanoparticles was added to the elastomer at a time and mixed for 1 min at 2500 rpm in a dual rotation mixer (SpeedMixer, Hauschild). This was repeated until a desired concentration was reached. The mixture was left to rest overnight and mixed again in order to reach a homogenous texture that was kept as a stock for the following steps. To create the gasket ink, Sylgard 184 with 15% fumed silica was supplemented with 20% Dowsil SE 1700 (Dow Corning). To create the compartment ink, Dowsil SE 1700 was supplemented with 10% Sylgard 184. Corresponding crosslinkers for the elastomers were added in 10:1 ratio. The inks were mixed for 30 s at 2700 rpm in a dual rotation mixer and loaded in a 5 cc syringe barrels. In order to remove the bubbles, syringes were centrifuged for 3 min at 1700 G. Syringes were then capped with plastic conical needles (200 µm inner diameter) and loaded onto the 3D Discovery bioprinter (regenHU). Silicon wafers containing the master mold were immobilized on the printing stage using vacuum suction. Printing path, based on the design created for the master fabrication, was defined in BioCad software (regenHU). Printing was done at room temperature directly on the silicon wafer via pneumatic extrusion with the gasket ink extruded at 550 Pa and the compartment ink at 275 Pa air pressure. Nozzle speed for both inks was set at 10 mm s^−1^. Gasket ink was printed at a 150 µm layer height while the compartment ink was printed at a 190 µm layer height. For the devices with PMMA or PLA extensions, only three layers of compartment ink were printed in order to allow a flat surface to be present for the attachment of fibers. Following the printing, the silicon wafer containing the printed devices was placed in the oven at 60 °C for 2 days in order to fully cure silicon elastomers. PLA extensions were printed on a Felix Pro 3 3D printer (Felix Robotics) using a transparent PLA filament. PMMA extensions were micromilled in a 5 mm thick PMMA sheet using a 1.5 mm endmill. Extensions were attached to the base by applying a thin layer of PDMS elastomer and curing the device overnight at 60 °C. Once the curing was over, individual devices were peeled off the silicon wafer and sterilized in 96% ethanol. Each device was bound covalently to a glass coverslip by exposure to air plasma. As a final step before the cells were seeded, compartments were incubated at 37 °C overnight with 1:100 dilution of Geltrex (ThermoFisher Scientific) in phosphate‐buffered saline (PBS) in order to facilitate cell adhesion to the glass surface. Videos and photographs of the printing process and the final devices were taken using a D7200 camera (Nikon). Brightfield images of the wall cross‐section and microchannel designs were taken on a Primovert inverted widefield microscope (Zeiss).

##### Rheology

Characterization of the rheological properties of the inks was performed on a Discovery HR‐2 rheometer (TA Instruments) equipped with a 25 mm parallel‐plate geometry. Measurements were taken at 25 °C on freshly mixed inks. Oscillatory amplitude sweeps at 1 Hz were performed for the values of strains ranging from 0.01% to 100%. Same amplitude sweeps were used to obtain complex viscosity values at 0.01% strain. Steady‐state flow curves were acquired at shear rates varying between 0.01 and 100 s^−1^.

##### MALDI–TOF Oligomer Detection

15 conventional devices were prepared per condition (2 h curing time and 24 h curing time). Each microfluidic channel in a device was filled with 10 µL of ultrapure water and incubated for 72 h at 37 °C. The contents of all the channels were pooled together, lyophilized, and resuspended in toluene. Dithranol matrix, sample, and silver trifluoroacetate were mixed in 10:5:1 ratio and analyzed on a MALDI–TOF mass spectrometer (Autoflex Speed, Bruker). 0.02 mg mL^−1^ 10 cs PDMS in toluene was used as a reference sample.

##### hNSC Culture and Differentiation

Both hNSC lines used in this study were cultured identically as described before.^[^ ^19^ ^]^ The hNSCs were cultured for at least two passages after thawing before being seeded onto the coated multicompartment devices. Cells were seeded in the same medium that was used before for their maintenance, termed growth medium. The basis of this growth medium is DMEM/F12 (ThermoFisher Scientific) with GlutaMAX (ThermoFisher Scientific). Additionally, the medium contained 6 g L^−1^ glucose (Sigma Aldrich), 5 × 10^−3^ m HEPES (ThermoFisher Scientific), 0.5 % w/v AlbuMAX (ThermoFisher Scientific), 40 × 10^−6^ m each of l‐alanine (MerckMillipore), l‐asparagine monohydrate (MerckMillipore), l‐aspartic acid (MerckMillipore), l‐glutamin acid (MerckMillipore), and l‐proline (MerckMillipore), 100× diluted N‐2 supplement (ThermoFisher Scientific), penicillin/streptomycin mix, 20 ng L^−1^ each of epidermal growth factor (EGF) and fibroblasts growth factor (FGF) (R&D systems).

24 h after seeding, the growth medium was replaced with differentiation medium. Differentiation medium was prepared using the same components as growth media, but with the EGF and FGF being substituted by 1 × 10^−3^ m dibutyryladenosine 3′,5′‐cylic monophosphate sodium salt (Sigma Aldrich) and 2 ng L^−1^ GDNF (PeproTech). After 48 h, the differentiation medium was completely replaced with fresh differentiation medium. Then, 2/3 differentiation medium was replaced with fresh differentiation medium every second day until the end of the experiment.

##### Generation of Induced Neurons from Fibroblasts

Adult dermal fibroblasts were obtained from the Parkinson's Disease Research clinic at the John van Geest Centre for Brain Repair (Cambridge, UK) with informed consent from the patients and used under local ethical approval (REC 09/H0311/88). For biopsy sampling information see Drouin‐Ouellet et al. 2017.^[^ ^26^ ^]^ Fibroblasts expansion and cultures see Birtele et al. 2019.^[^ ^37^ ^]^ The cell lines used in this study came from two healthy 67‐ and 70‐year old females at the time of skin sampling.

The reprogramming protocol used DNA plasmids expressing the open reading frames (ORFs) for Ascl1, Brn2 with miRNA loops for miR‐9/9* and miR‐124 in combination with shRNA targeting REST. Used lentiviruses were third generation vectors containing a nonregulated ubiquitous phosphoglycerate kinase (PGK) promoter. The vectors were used at final MOI 20. All viruses used in this study tittered between 3 × 10^8^ and 6 × 10^9^ pfu mL^−1^.

Prior to plating, the wells were coated overnight with a combination of polyornithine (15 µg mL^−1^), fibronectin (0.5 ng µL^−1^), and laminin (5 µg mL^−1^) (PFL). Fibroblasts were plated at a density of 27 800 cells cm^−2^. Three days after the viral transduction, the fibroblast medium was replaced with neural differentiation medium (NDiff227, Takara‐Clontech) supplemented with growth factors at the following concentrations: LM‐22A4 (2 × 10^−6^ m, R&D Systems), GDNF (2 ng mL^−1^, R&D Systems), NT3 (10 ng µL^−1^, R&D Systems), as well as db‐cAMP (0.5 × 10^−3^ m, Sigma) and the small molecules CHIR99021 (2 × 10^−6^ m, Axon), SB‐431542 (10 × 10^−6^ m, Axon), noggin (0.5 µg mL^−1^, R&D Systems), LDN‐193189 (0.5 × 10^−6^ m, Axon), valproic acid sodium salt (VPA, 1 × 10^−3^ m , Merck Millipore). Half medium changes were performed every two days for the first 30 days of conversion, whereas in the later stages of conversion the medium changes were done every three days. At 18 days post‐transduction, the small molecules were withheld, and the neuronal medium was supplemented only with LM‐22A4, GDNF, NT3, and db‐cAMP until the end of the experiment.

##### Whole Cell Patch‐Clamp Recording

Cells were moved to a recording chamber and submerged in a flowing artificial cerebrospinal fluid (ACSF) solution gassed with 95% O_2_ and 5% CO_2_ at 23 °C according to standard procedures for recording. The composition of the ACSF was: 126 ×10^−3^ m NaCl, 2.5 ×10^−3^ m KCl, 1.2 ×10^−3^ m NaH_2_PO_4_‐H_2_O, 1.3 ×10^−3^ m MgCl_2_‐6H_2_O, 2.4 ×10^−3^ m CaCl_2_‐6H_2_O, 22 ×10^−3^ m NaHCO_3_, 10 ×10^−3^ m glucose. The pH of the solution was adjusted to 7.4. Multi‐clamp 700B (Molecular Devices) was used for recordings and signals were acquired at 10 kHz using pClamp10 software and a data acquisition unit (Digidata 1440A, Molecular Devices). Input resistances and injected currents were monitored throughout the experiments. Borosilicate glass pipettes (4–7 MΩ) for patching were filled with the following intracellular solution: 122.5 ×10^−3^ m potassium gluconate, 12.5 ×10^−3^ m KCl, 0.2 ×10^−3^ m EGTA, 10 ×10^−3^ m HEPES, 2 ×10^−3^ m MgATP, 0.3 ×10^−3^ m Na_3_GTP, and 8 mv NaCl. Solution was adjusted to pH 7.3 with KOH. Resting membrane potentials were monitored immediately after breaking‐in in the current‐clamp mode. Membrane capacitance was monitored throughout the recordings and recordings where discarded if changes in values higher than 30% were observed. The membrane potential was kept between −60 to −70 mV and currents were injected from −20 to +90 pA with 10 pA increments to induce AP. Inward sodium and delayed rectifying potassium currents were measured in the voltage clamp mode at depolarizing steps of 10 mV.

##### Calcium Imaging—GCamP3

The MAP2‐GCamP3 lentiviral reporter plasmid (Addgene Plasmid 43917) was packaged in HEK‐293T cells using pM2.G and PMDLg. Lentivirus titers between 3 × 10^8^ and 6 × 10^9^ pfu mL^−1^ were used for these experiments. Virus was added to the media at day 18 of culture at MOI 5 to ensure detectable fluorescent levels. After 24 h incubation, a total media change was performed and fluorescent neurons for analysis appeared 24–48 h after transduction.

##### Calcium Imaging—Fluo3 AM

Cells were incubated for 30 min at 37 °C with 3 × 10^−6^ m Fluo3 AM calcium indicator (ThermoFisher Scientific) in cell culture medium containing 0.02% Pluronic F‐127 (Sigma Aldrich). Cells were then rinsed in cell culture media and incubated for 30 min at 37 °C before imaging.

Calcium imaging of neurons containing the MAP2‐GCamP3 reporter and the cells loaded with Fluo3AM was performed in the same way, as follows. Cell culture media was replaced with 100 µL baseline buffer containing 1.2 × 10^−3^ m MgCl_2_, 2 × 10^−3^ m CaCl_2_, 150 × 10^−3^ m NaCl, 5 × 10^−3^ m KCl, 5 × 10^−3^ m glucose, and 10 × 10^−3^ m HEPES. Imaging was performed on an inverted Ti2 microscope (Nikon) equipped with a CSU‐W1 spinning disc system (Yokogawa), a sCMOS camera (Teledyne Photometrics), and a 20× objective. Environment control chamber was used to maintain the temperature at 37 °C and CO_2_ level at 5% during imaging. Exposure time was set to 30 and 100 ms depending on the dynamics of calcium transients. Firstly, spontaneous activity was recorded. Finally, stimulated calcium influx was recorded by inducing cell membrane depolarization through the injection of 50 µL of stimulation buffer containing 1.2 × 10^−3^ m MgCl_2_, 2 × 10^−3^ m CaCl_2_, 5 × 10^−3^ m NaCl, 450 × 10^−3^ m KCl, 5 × 10^−3^ m glucose, and 10 × 10^−3^ m HEPES buffer. Images were analyzed in ImageJ (NIH) and plotted in QtiPlot.

##### Immunocytochemistry and Live/Dead Staining

At the experimental endpoint, cells were fixed with 4% paraformaldehyde and stained using standard protocols and the following antibodies: *β*‐tubulin III (mouse, Sigma Aldrich T8660, 1:1000), TH (rabbit, PelFreez Biologicals P40101, 1:1000), GFAP (rabbit, Dako Z0334, 1:1000; mouse, BiolegendSM121, 1:500), synapsin (rabbit, Invitrogen 72732524, 1:2000). For fluorescent stainings, Alexa 546 (goat anti‐mouse, Thermofisher Scientific A‐11030, 1:500), Alexa 647 (goat anti‐rabbit, ThermoFisher Scientific A‐21245, 1:500), Cy5 (donkey anti‐rabbit, Jackson Immunoresearch, 711605152, 1:500). Nuclei were counterstained using DAPI (4′,6‐diamidino‐2‐phenylindole) (1:1000) or 2 µg mL^−1^ Hoechst 33342.

Live and dead staining was done by incubating cells for 30 min at 37 °C in 2 × 10^−6^ m Calcein AM (Sigma Aldrich) and 2 × 10^−6^ m Ethidium homodimer‐1 (EthD‐1, ThermoFisher).

The middle image in Figure 2d was acquired via a 5 × 5 cm tile scan with online image stitching on the above mentioned Ti2 microscope using an objective with 4× magnification. The rest of the images were acquired on an LSM 710 (Carl Zeiss) inverted confocal microscope. Objectives with 10×, 20×, and 60× magnification were used. Images were processed and analyzed in ImageJ software.

##### Scanning Electron Microscopy

Samples were mounted on 25 mm aluminum stubs, sputter coated with 10 nm platinum/palladium (80:20) and imaged in a Jeol JSM‐7800F scanning electron microscope.

##### Statistical Analysis

Microsoft Excel and QtiPlot were used for the statistical analysis of the data presented in this work. The pre‐processing and data presentation were done in the following way. Figure 1c,d shows mean ± SD. Data in Figure 5b,c is represented as time series of total fluorescence intensity from selected regions of interest differential to the initial value. Data in Figure 6b shows the total number of neurites counted in the compartment adjacent to the compartment with cell bodies. Color coding indicates the length of the neurite binned in six categories. The data in Figure S3c in the Supporting Information underwent zero mean normalization and is presented as mean ± SD with individual data points also displayed. Figure S3d in the Supporting Information shows mean ± SD with individual data points displayed as well. Sample sizes are noted in the corresponding figures.

## Conflict of Interest

The authors declare no conflict of interest.

## Supporting information

Supporting Information

Supplemental Video 1

Supplemental Video 2
