# Supplementary material for: 3D‐Printed Soft Lithography for Complex Compartmentalized Microfluidic Neural Devices
Source: Adv Sci (Weinh). 2020 Jun 15;7(16):2001150. doi: 10.1002/advs.202001150 (PMC7435242; doi:10.1002/advs.202001150)

Supplementary Information

3D-Printed Soft Lithography for Complex Compartmentalized Microfluidic Neural Devices

Janko Kajtez, Sebastian Buchmann, Shashank Vasudevan, Marcella Birtele, Stefano Rocchetti, Christian Jonathan Pless, Arto Heiskanen, Roger A. Barker, Alberto Martínez-Serrano, Malin Parmar, Johan Ulrik Lind*, Jenny Emnéus*

Dr. J. Kajtez, M. Birtele, Prof. M. Parmar
Department of Experimental Medical Sciences, Wallenberg Neuroscience Center, Division of Neurobiology and Lund Stem Cell Center, BMC A11, Lund University, S-22184, Lund, Sweden
e-mail: janko.kajtez@med.lu.se

S. Buchmann, S. Vasudevan, S. Rocchetti, Dr. A. Heiskanen, Prof. J. Emnéus
Department of Biotechnology and Biomedicine (DTU Bioengineering), Technical University of Denmark, Produktionstorvet, Building 423, 2800 Kgs. Lyngby, Denmark

Prof. R. A. Barker
John van Geest Centre for Brain Repair & Department of Neurology, Department of Clinical Neurosciences and WT-MRC Cambridge Stem Cell Institute, University of Cambridge, Cambridge, United Kingdom

Prof. A. Martínez-Serrano
Department of Molecular Biology, Universidad Autónoma de Madrid, and Department of Molecular Neuropathology, Center of Molecular Biology Severo Ochoa (UAM-CSIC). Nicolás Cabrera 1, 28049 Madrid, Spain

C. J. Pless, Dr. J. U. Lind
Department of Healthcare Technology (DTU Health Tech), Technical University of Denmark, Produktionstorvet, Building 423, 2800 Kgs. Lyngby, Denmark
e-mail: joli@dtu.dk

Figure S1 *Additional device designs:* **a)** Concentric circles device bound to a glass slide with colored compartments. **b)** Brightfield image showing the array of microchannels on the bottom face of the compartment wall. **c)** Higher magnification brightfield image displaying the "return-to-sender" microchannel design. **d)** Axotomy device bound to a glass slide with colored compartments. **e)** Brightfield image showing the array of microgrooves on the bottom face of the walls; left and right compartments are open compartments for cell seeding; middle compartment is a closed microfluidic compartment (180 μm height) where the axons are projected and could be severed by vacuum suction. **f)** Higher magnification brightfield image displaying the directional microchannel design.

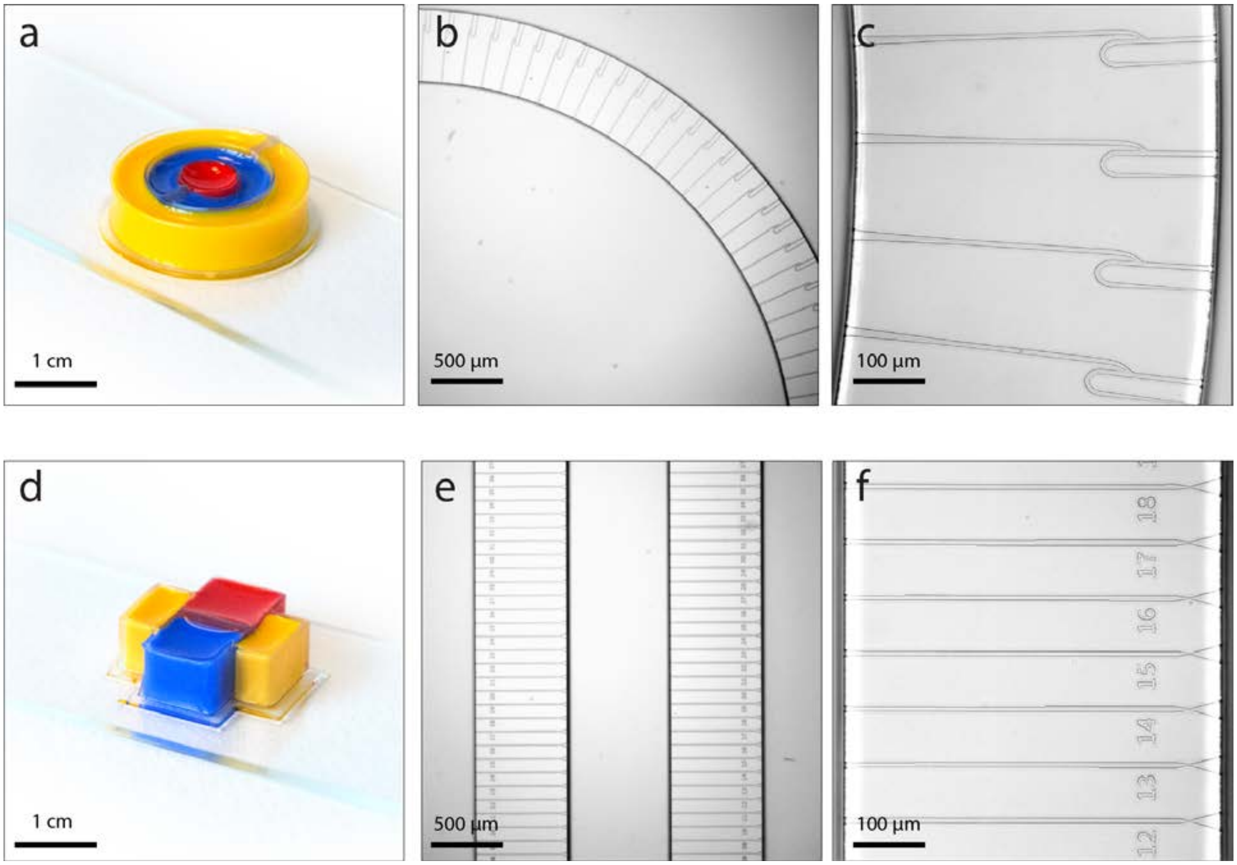

Figure S2 *Issues related to manual postprocessing:* Cutting out open compartments with a scalpel or a biopsy punch after bulk PDMS casting is a task highly dependent on the dexterity of the person performing it. In order to preserve the architecture of the microchannels in compartmentalized microfluidic devices and create vertical compartment walls at the open ends of the microchannels, a micrometer precision is needed. Electron microscopy images of PDMS devices after cutting with a scalpel illustrate what issues misalignment of only 200-300 μm can cause. Cutting line is labelled by yellow arrows. **a)** Cutting away from the microchannel entrance creates overhanging structures that prevent uniform cell seeding next to the microchannels resulting in low neuronal density in the same area. **b)** Cutting into the microchannels results in microchannels with reduced non-uniform length. **c)** Cutting into the microchannels destroys any microarchitecture design that was determined for the entrance/exit of the microchannels.

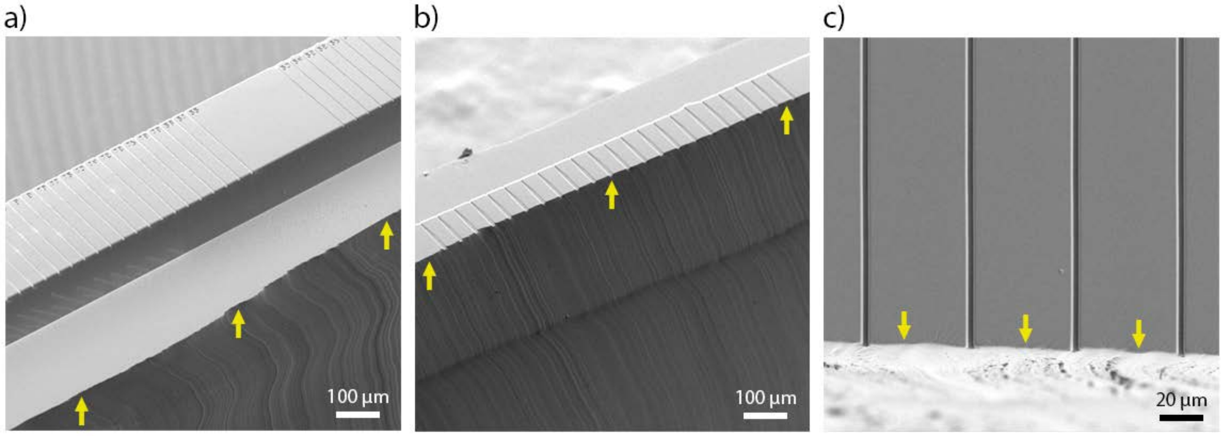

Figure S3 *Improved accuracy and reproducibility of microcontact printing stamp fabrication:* In order to showcase the advantages of the additive manufacturing approach, we compared accuracy and reproducibility of the conventional soft lithography with 3D-printed soft lithography for the fabrication of PDMS microcontact printing stamps. In order to determine the final position of the printed pattern on a substrate, it is important to know the position of the pattern on the stamp with respect to the outer borders of the stamp. Therefore, reproducibility and accuracy in the fabrication of the stamps would result in a more precisely positioned microcontact printing. The results shown in this figure indicate that 3D printing approach yields more accurate and reproducible stamps **a) b)** Photographs on the top show the individual stamps with square base 6 x 6 mm in dimension. SEM images display the pattern of interest (white arrow) and device edges (red arrows). **c)** Measurements of the offset with zero mean normalization of the stamp edges from the pattern of interest. 3D printed stamps have 5 times smaller standard deviation. **d)** Measurements of the tilt angle of the stamp edges with respect to the lateral axis of the pattern. There is a 10-fold decrease in the standard deviation for the 3D printing approach in comparison with the conventional soft lithography. In both offset and tilt plots mean is indicated by a colored line while standard deviation is indicated by a colored box.

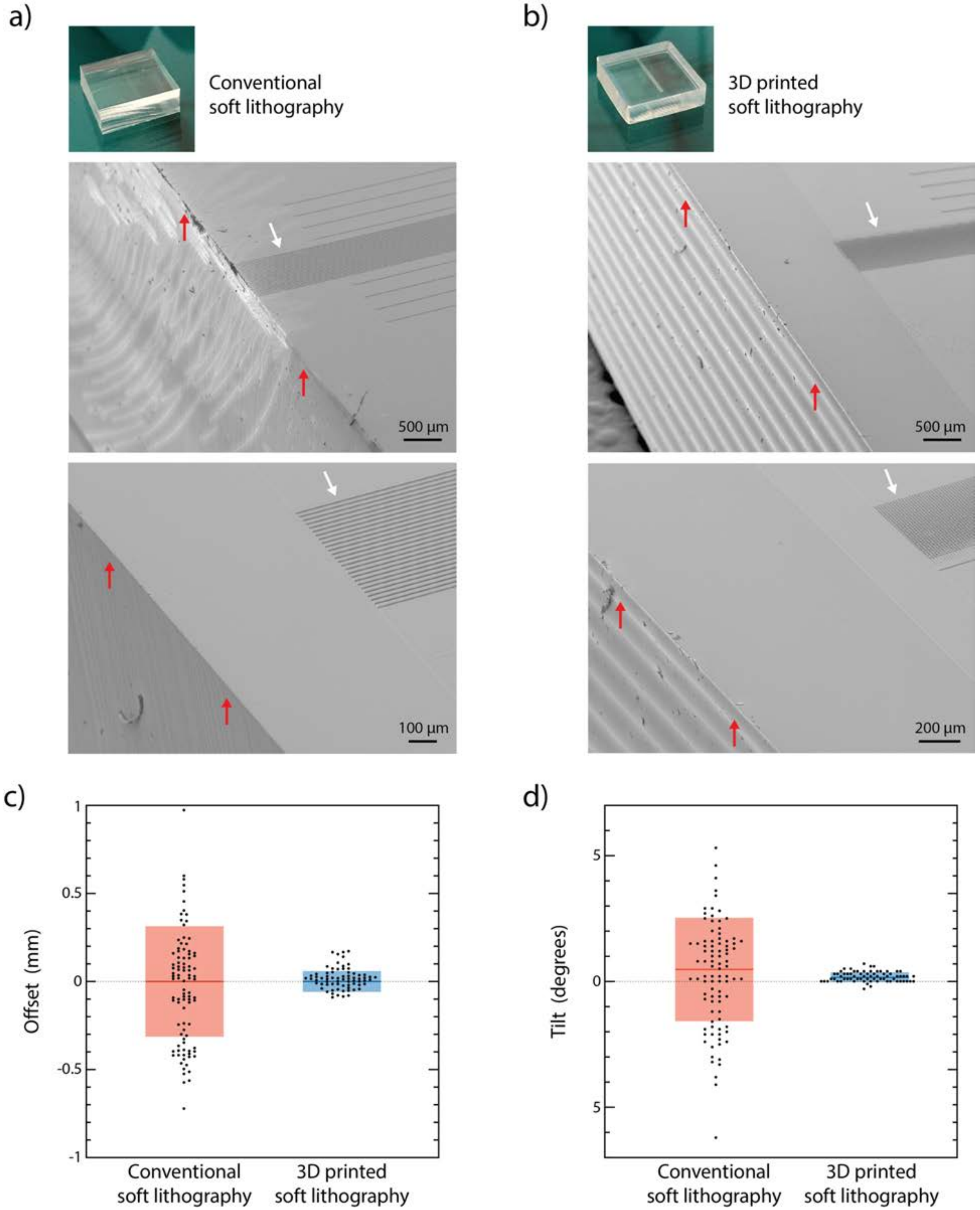
a)
Conventional
soft lithography
500 µm
100 µm
b)
3D printed
soft lithography
500 µm
200 µm
c)
Offset (mm)
1
0.5
0
-0.5
-1
Conventional
soft lithography
3D printed
soft lithography
d)
Tilt (degrees)
5
0
5
Conventional
soft lithography
3D printed
soft lithography

Figure S4 *hNSC differentiation in XonChips^{TM}:* Immunocytochemistry showing successful differentiation of hNSCs in commercial microfluidic devices made from cyclin olefin copolymer. a) M-hNSCs stained for nuclei (blue), neuronal marker ß-III tubulin (green), and tyrosine hydroxylase (red). b) F-hNSCs stained for nuclei (blue) and astrocyte marker GFAP (green).

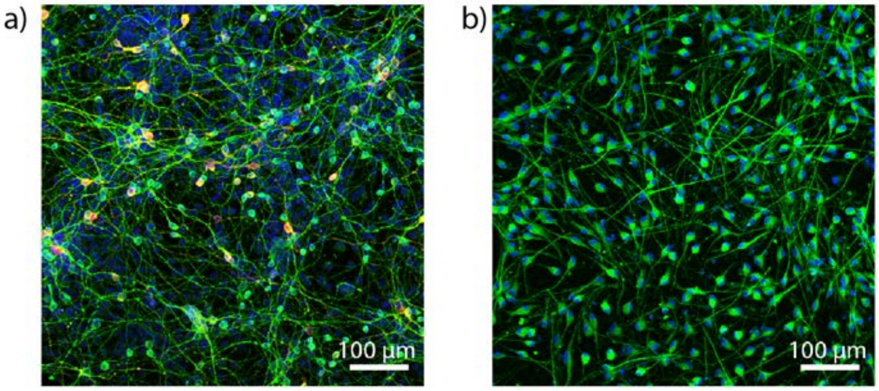

Figure S5 *Acute toxicity:* Both images show F-hNSCs at day 14 of differentiation in a commercial 24 well plate. Differentiation media given to the cells on the left was preconditioned in PDMS wells. Necrotic region, as observed in the conventional PDMS devices, is evident in the top half of the image. On the right, control cells that were given fresh media show no sign of necrosis.

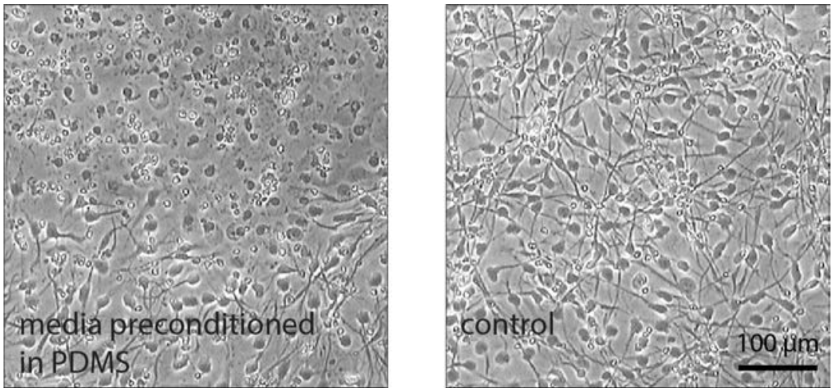

Figure S6 *MALDI-TOF detection of uncured PDMS oligomers:* **a)** Control sample with 0.02 mg/ml 10 cs PDMS. **b)** Detectable oligomers in microchannel media from conventional devices cured for 2h at 60 ˚C recognized by 74 Da separation between the peaks. **c)** No oligomers are detected in microchannel media from conventional devices cured for 24h at 60 ˚C. **d)** Negative control measurement from the matrix alone.

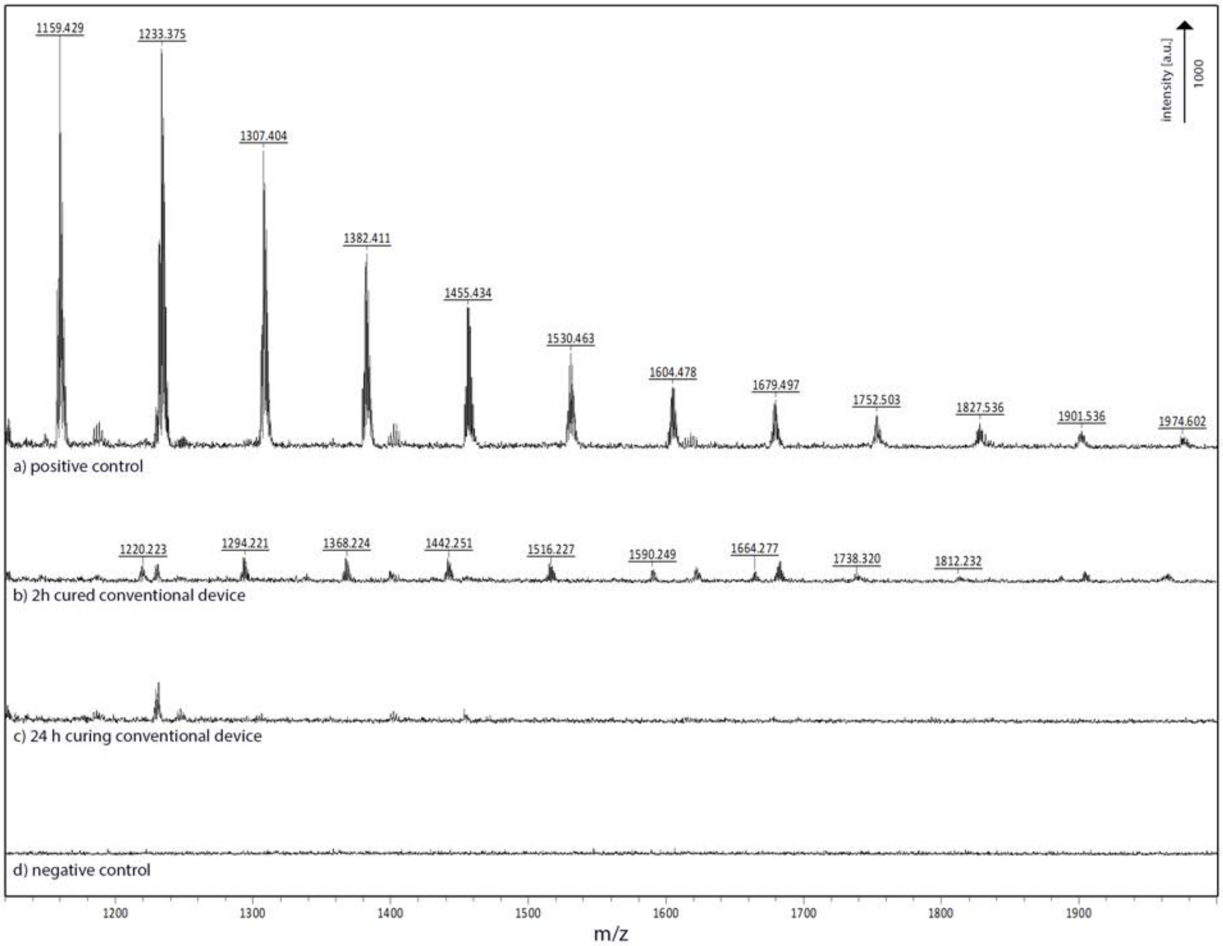

Figure S7 *Comparison between devices constructed from different materials:* Conventional device is fabricated by pouring PDMS over a mold, cutting out the individual chips with a scalpel and inlets/outlets with a biopsy punch. PDMS-PDMS device is fully printed using 3D-printed soft lithography while the last two devices are extended with PMMA and PLA compartment walls. All the devices are covalently bound to glass substrates and each compartment is filled with a dye for visualization.

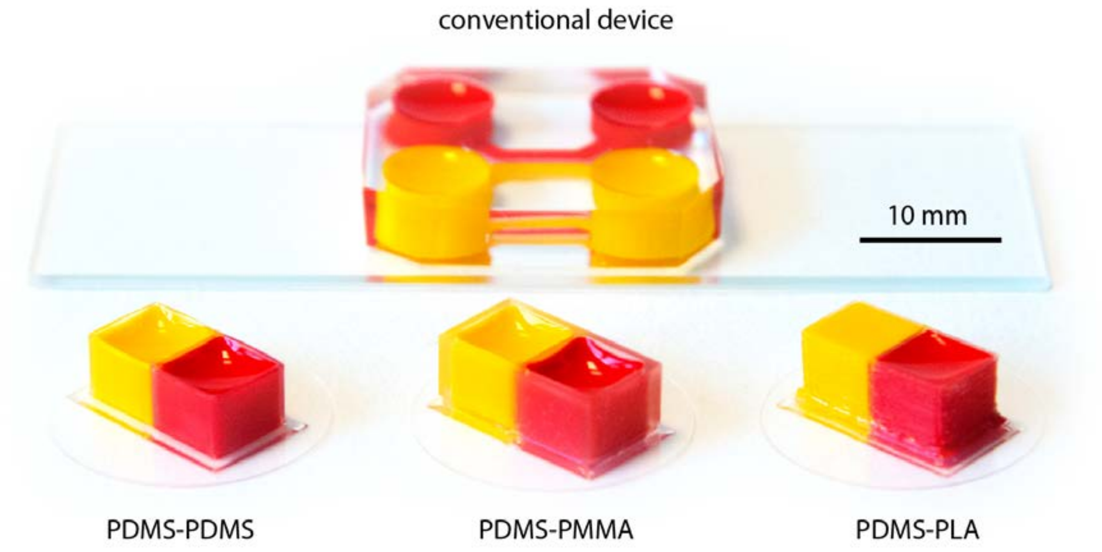

Figure S8 *Fabricating devices using an alternative elastomer:* A side and frontal view of a fully 3D printed compartmentalized neural device based on styrene-isoprene-styrene (SIS) block co-polymer inks (a). *Inks for well structures:* 1:2:3 (w:w:w) SIS (Sigma-Aldrich 432493) : Butyl Acetate : Hollow SiO2 glass spheres (particle size 9-13µm - Sigma-Aldrich 440345), *Gasket ink:* 1:3 (w:w) SIS : Butyl Acetate. Printed using 410µm and 200µm nozzles, respectively. Cured for 48 hours at 60°C after printing. Confocal images show live (green) and dead (red) staining of differentiated M-hNSCs (b) and F-hNSCs (c) after 40 days in the devices. Characteristic morphological changes for the respective cell lines. While majority of cells in the generated network are alive, there is a number of dead cells and cells with unhealthy morphology. Further, the lower adhesion of SIS devices to glass, as compared to that of PDMS, hindered faithful capture of micro-channels between compartments.

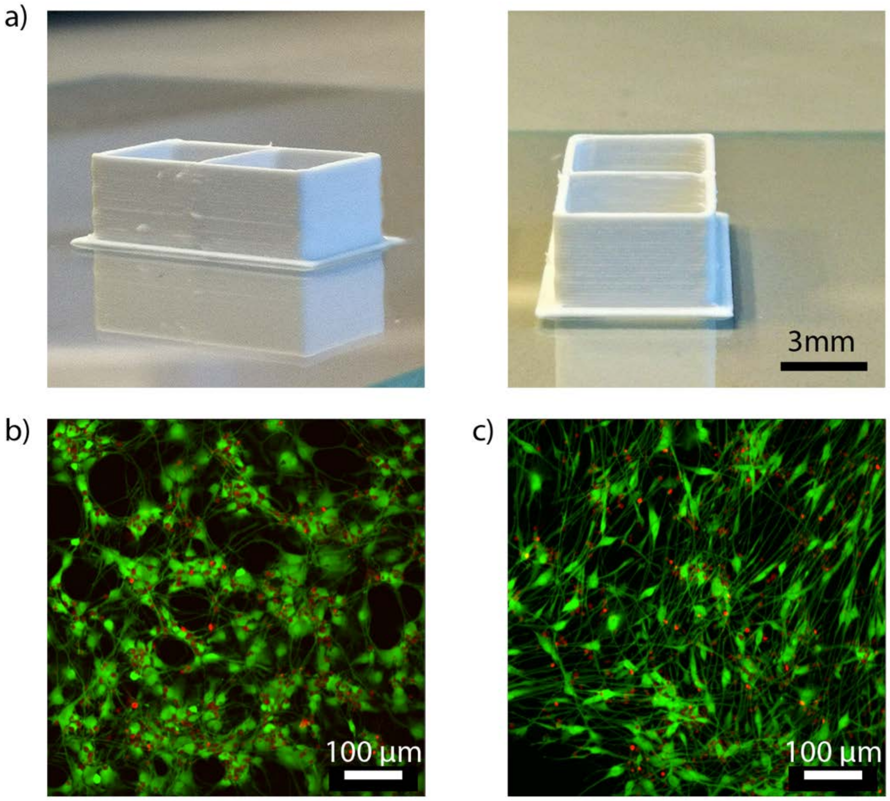

Figure S9 *Generation of induced neurons from adult human fibroblasts:* Vimentin was used to stain fibroblasts. Map2 and Tau were used to stain microtubule associated proteins in neuronal cells. DAPI was used to stain cell nuclus. Double positive MAP2 and TAU cells show successful conversion of a subpopulation of adult fibroblasts into a neuronal lineage.

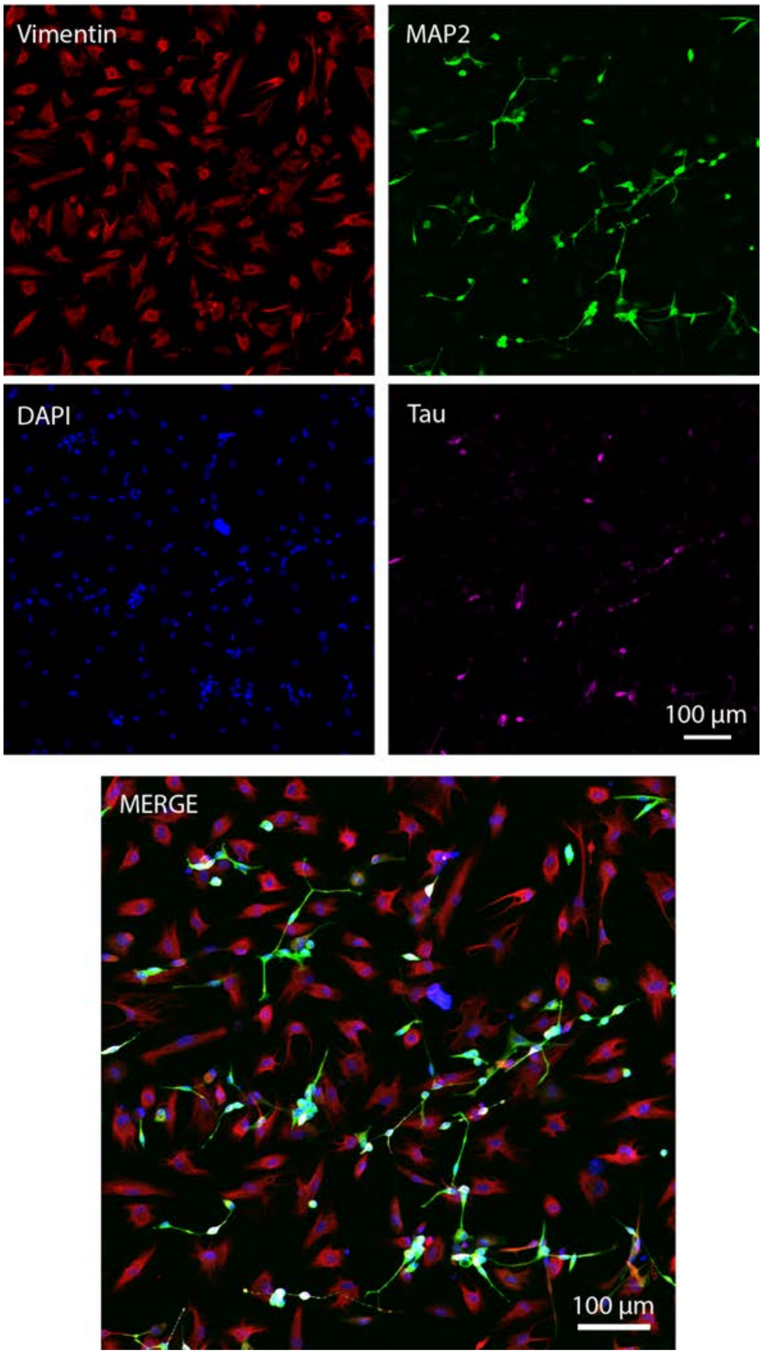

Figure S10 *Neurite growth through 900 µm long microchannels:* Brightfield image on the left shows straight microchannels between two compartments. Middle image indicates cell nuclei counterstained with DAPI. Right image, with ß-tubulin staining neurons, illustrates the inability of neuronal projections to transverse the whole channel length within 20 days of differentiation.

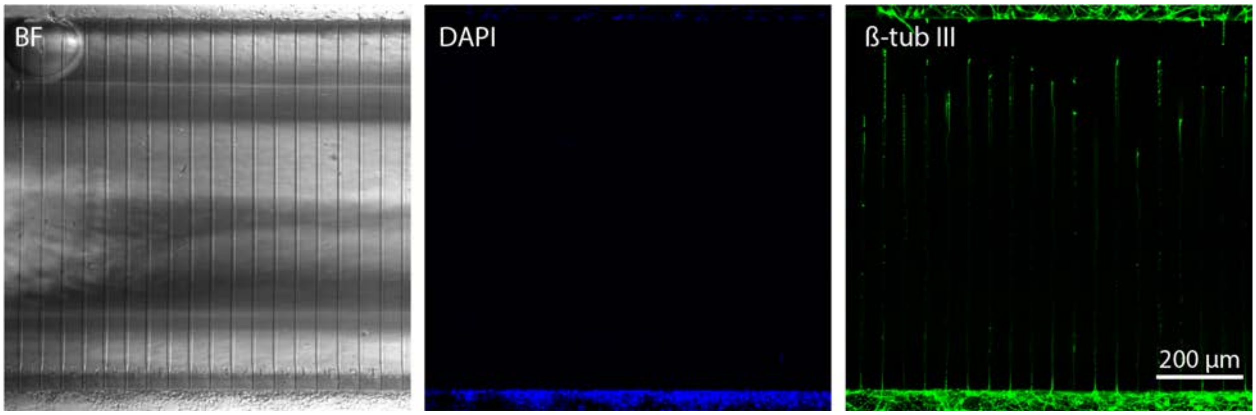

Figure S11 *Differentiated F-hNSCs do not project into adjacent compartment:* Fluorescence images showing differentiated F-hNSCs seeded in the lower compartment. No projections are identified in the adjacent compartment indicating that these cells are not able to extend through the whole length of microchannels (450 µm).

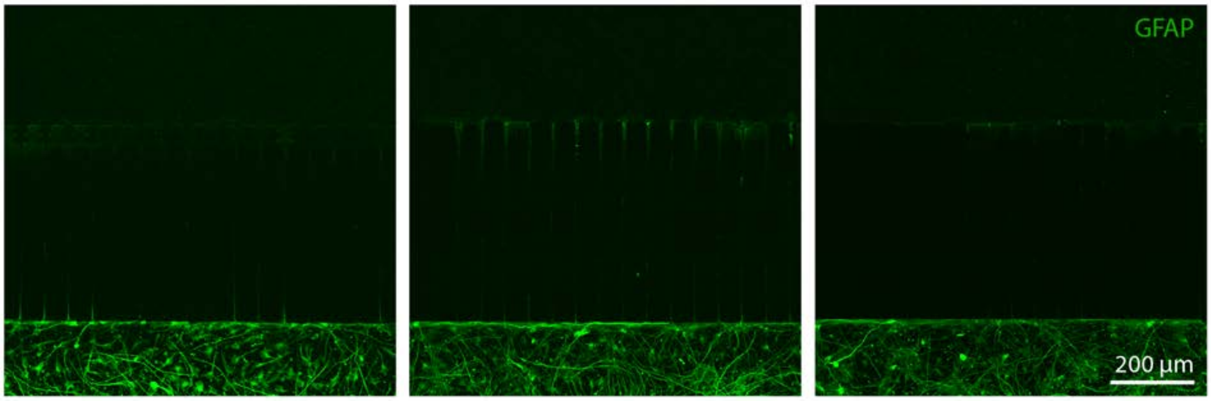

Video 1 *3D-printing process:* The video shows the printing process of the "brain" chip at 20x accelerated speed. First a layer of the gasket ink is printed (blue nozzle highlighted). Afterwards the compartment ink is printed to create compartment walls (red nozzle highlighted). Time is marked as mm:ss.

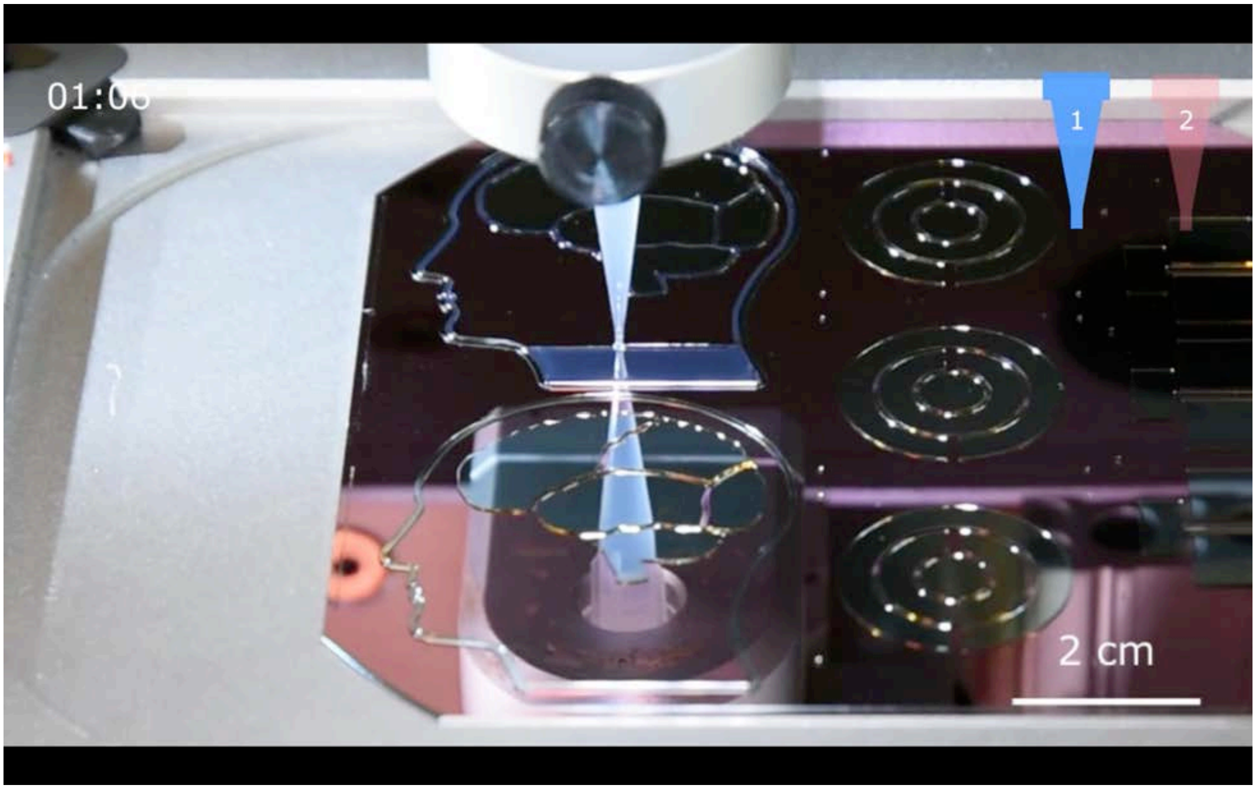

Video 2 *Calcium influx imaging of chemically stimulated neuronal activity:* The video shows confocal fluorescence imaging of calcium influx in neurons generated from M-hNSCs. Membrane depolarization was induced by an addition of the stimulation buffer.

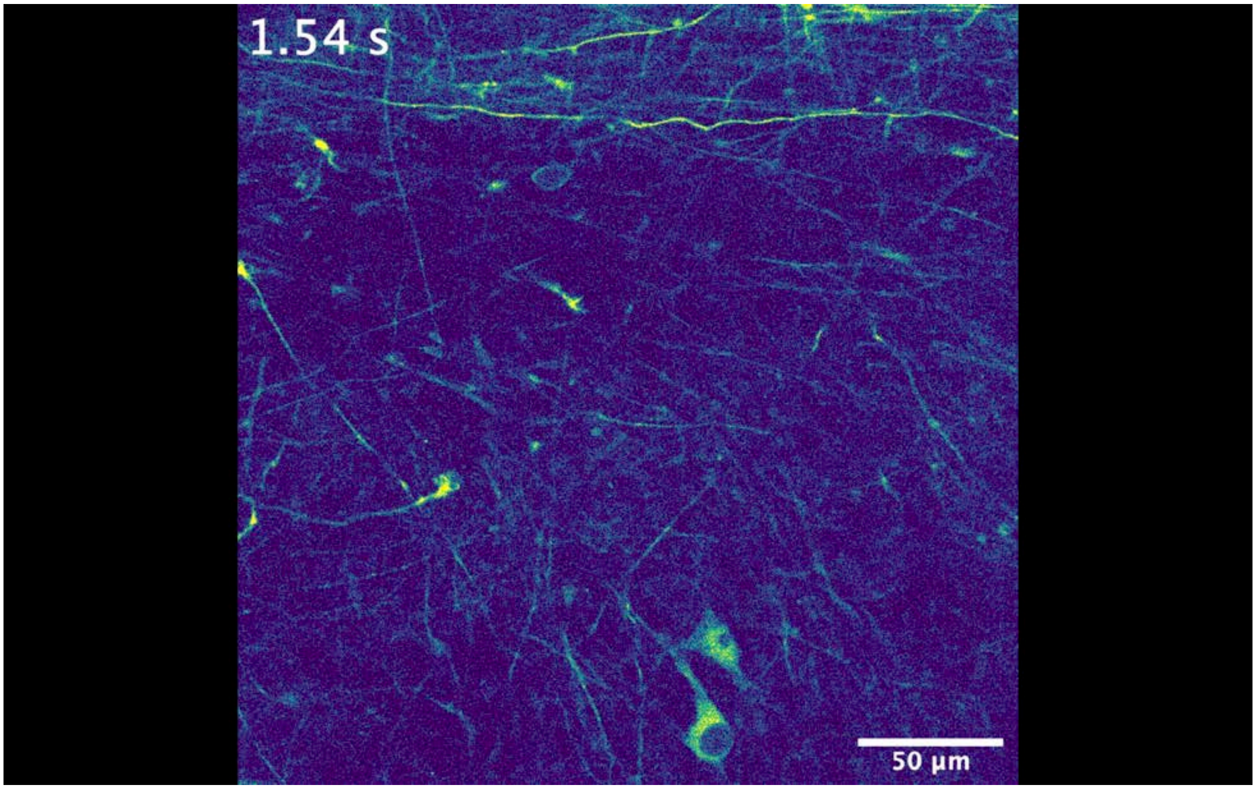

Supplement: Supplementary file 1 — Supporting Information [file ADVS-7-2001150-s001.pdf]
